# A preliminary study of synthetic magnetic resonance imaging in rectal cancer: imaging quality and preoperative assessment

**DOI:** 10.1186/s13244-021-01063-w

**Published:** 2021-08-21

**Authors:** Li Zhao, Meng Liang, Pu-yeh Wu, Yang Yang, Hongmei Zhang, Xinming Zhao

**Affiliations:** 1 Department of Diagnostic Radiology, National Cancer Center/National Clinical Research Center for Cancer/Cancer Hospital, Chinese Academy of Medical Sciences and Peking Union Medical College, No. 17, Panjiayuan Nanli, Chaoyang District, Beijing, 100021 China; 2 GE Healthcare, MR Research China, No. 1 Tongji South Road Beijing Economic Technology Development Area, Beijing, 100176 China

**Keywords:** Rectal cancer, Magnetic resonance imaging, Synthetic imaging, Evaluation study

## Abstract

**Purpose:**

To compare the imaging quality, T stage and extramural venous invasion (EMVI) evaluation between the conventional and synthetic T2-weighted imaging (T2WI), and to investigate the role of quantitative values obtained from synthetic magnetic resonance imaging (MRI) for assessing nodal staging in rectal cancer (RC).

**Methods:**

Ninety-four patients with pathologically proven RC who underwent rectal MRI examinations including synthetic MRI were retrospectively recruited. The image quality of conventional and synthetic T2WI was compared regarding signal-to-noise ratio (SNR), contrast-to-noise (CNR), sharpness of the lesion edge, lesion conspicuity, absence of motion artifacts, and overall image quality. The accuracy of T stage and EMVI evaluation on conventional and synthetic T2WI were compared using the Mc-Nemar test. The quantitative T1, T2, and PD values were used to predict the nodal staging of MRI-evaluated node-negative RC.

**Results:**

There were no statistically significant differences between conventional and synthetic T2WI in SNR, CNR, overall image quality, lesion conspicuity, and absence of motion artifacts (*p* = 0.058–0.978). There were no significant differences in the diagnostic accuracy of T stage and EMVI between conventional and synthetic T2WI from two observers (*p* = 0.375 and 0.625 for T stage; *p* = 0.625 and 0.219 for EMVI). The T2 value showed good diagnostic performance for predicting the nodal staging of RC with the area under the receiver operating characteristic, sensitivity, specificity, and accuracy of 0.854, 90.0%, 71.4%, and 80.3%, respectively.

**Conclusions:**

Synthetic MRI may facilitate preoperative staging and EMVI evaluation of RC by providing synthetic T2WI and quantitative maps in one acquisition.

**Supplementary Information:**

The online version contains supplementary material available at 10.1186/s13244-021-01063-w.

## Key Points

- Synthetic T2WI provides comparable image quality with the conventional T2WI.
- Synthetic T2WI enables similar diagnostic accuracy in local staging of rectal cancer.
- Synthetic MRI facilitate preoperative evaluation of rectal cancer by providing multiple images.

## Introduction

Colorectal cancer is the third most common malignancy worldwide, ranking third in mortality among female and male, respectively [1]. Rectal cancer (RC) accounts for approximately 30%–35% of colorectal cancer cases, which are mostly adenocarcinoma [2]. Magnetic resonance imaging (MRI), especially high-resolution T2-weighted imaging (T2WI), is recommended to be routinely used for assessing RC local stage, which is critical for treatment decisions and the prognosis prediction [3, 4].

Besides the conventional contrast-weighted imaging, quantitative relaxation mappings have been shown to play a certain role in identifying the tumor grade, metastatic lymph nodes, lymphovascular invasion, and therapeutic response of several tumors [5–9]. However, the separate acquisitions of quantitative mappings and contrast-weighted images can be time-consuming. Synthetic MRI, in which a multi-echo and multi-delay acquisition scheme is adopted, can be advantageous of shorting scanning time by simultaneously quantifying T1, T2 and proton density (PD) relaxometry and generating synthetic contrast-weighted images in a single scan [10, 11].

Synthetic MRI has been demonstrated to have excellent correlation with conventional mapping methods and comparable image quality to that of conventional contrast-weighted images in brain and knee [12–14]. Additionally, there have been some promising findings of synthetic MRI in various tumors, such as prostate cancer, breast cancer, bone metastasis [6, 15, 16]. Our previous study has proven that quantitative T1 and T2 values generated from synthetic MRI were useful for predicting prognostic factors of RC [17]. Another study showed that radiomics model based on synthetic MRI could improve the diagnostic performance of extramural venous invasion (EMVI) in rectal cancer [18]. To the best of our knowledge, the feasibility of the contrast-weighted imaging generated from synthetic MRI has not yet been reported.

Recently, the diagnostic accuracy and interobserver agreement of conventional nodal staging in RC remain unsatisfactory [19]. A previous study suggested that T2 value may be useful in differentiating metastatic lymph nodes in RC, but is limited by the node-by-node approach to match the lymph node on MRI with the pathological specimen [9]. Although previous studies have confirmed the associations between the characteristics of the primary tumor and the nodal staging, few studies have focused on the MRI-evaluated node-negative RC.

The aim of this study was to compare the image quality, T stage, and EMVI assessment between the conventional and synthetic T2WI, and to investigate the quantitative values for a more accurate nodal staging of MRI-evaluated node-negative RC.

## Materials and methods

### Participants

**Fig. 1:**
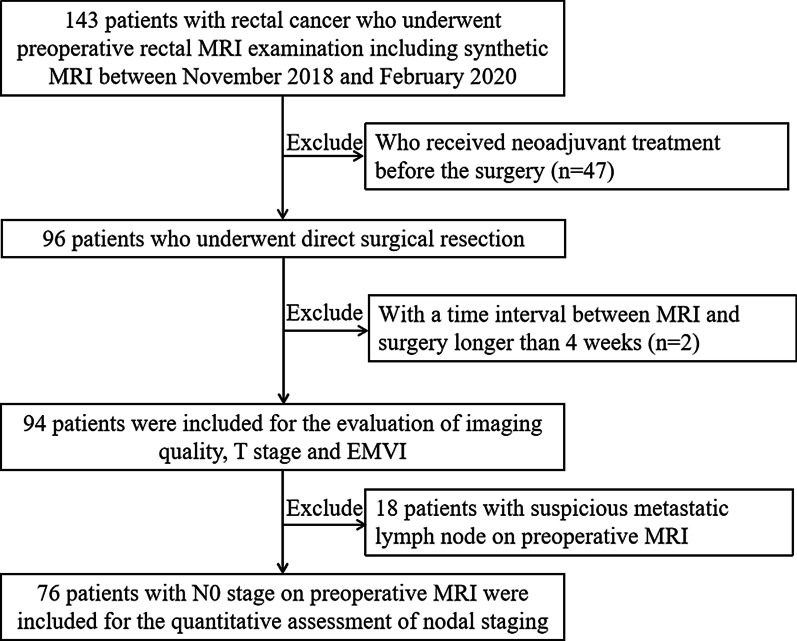
Flow chart of the study cohort

Our Institutional Review Board approved this retrospective single-center study and the informed consent was waived. In total, 143 patients with pathologically confirmed rectal adenocarcinoma who underwent rectal MRI examinations including the synthetic MRI sequence between November 2018 and February 2020 were enrolled. Exclusion criteria were as follows: (1) those who received neoadjuvant treatment before the surgery; (2) time interval between the MRI examination and surgery greater than 4 weeks. Finally, 94 patients were included in the study. Of these, 18 participants with suspicious metastatic lymph node on preoperative MRI were excluded from quantitative evaluation of nodal staging. The flowchart of the study cohort is shown in Fig. 1.

Pathological characteristics of surgical specimens were assessed according to the eighth edition of the American Joint Committee on Cancer (AJCC) TNM staging system [20].

### MRI acquisition

**Fig. 2:**
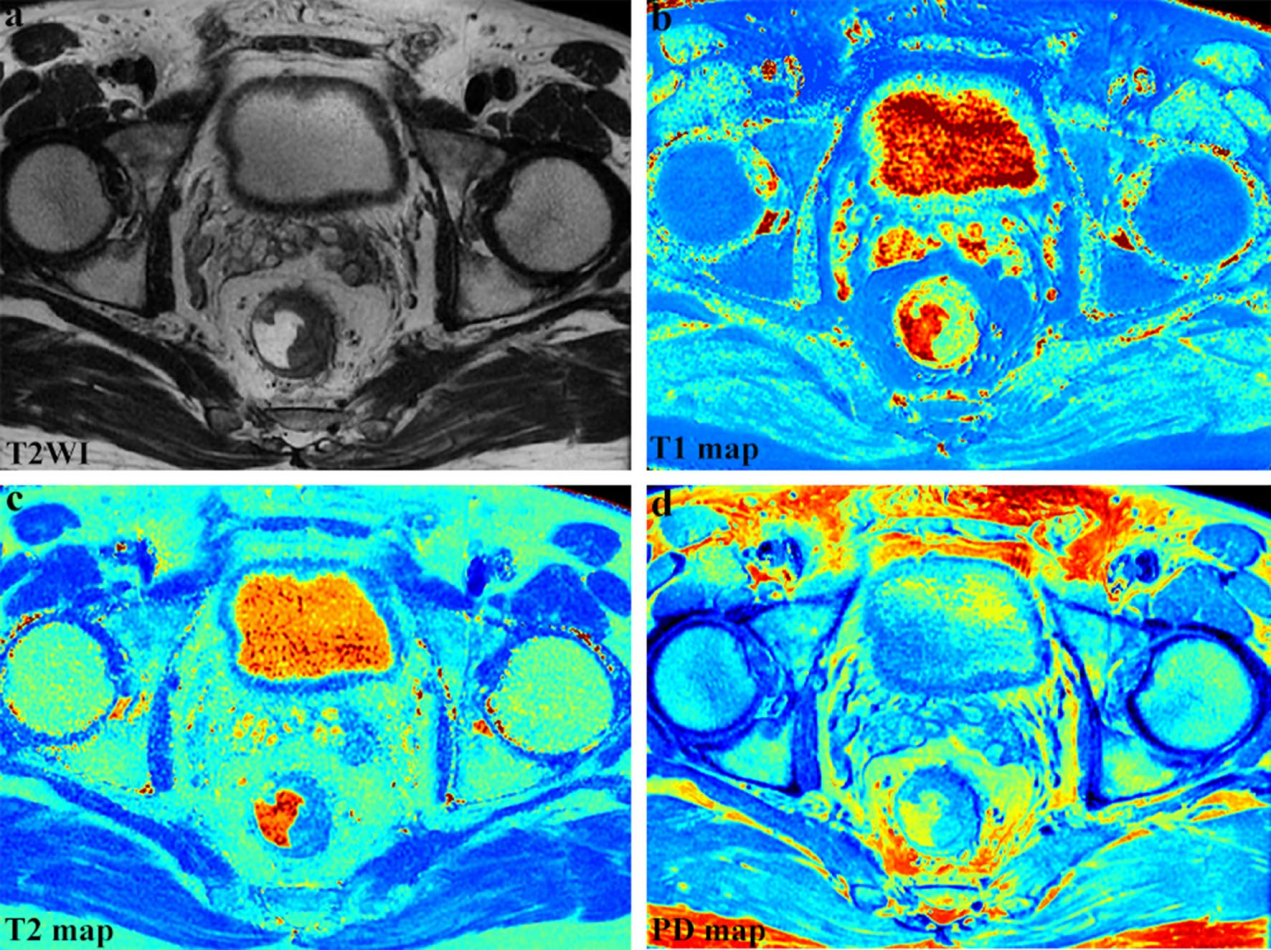
Representative synthetic magnetic resonance images from a 57-year-old male patient with rectal cancer. (**a**–**d**) T2-weighted imaging (T2WI), T1 map, T2 map, and PD map

All MRI examinations were performed on a 3.0 T scanner (SIGNA Pioneer, GE Healthcare, Milwaukee, WI) equipped with a 32-channel phased-array body coil. For bowel preparation, glycerin enema was used to empty the feces before the examination. Anisodamine hydrochloride (10 mg, except those with contraindications) was intramuscularly injected 15 min before the examination to reduce peristaltic artifacts. Axial T1-weighted imaging (T1WI), diffusion-weighted imaging (DWI) (b values of 0 and 1000 s/mm^2^), oblique axial/coronal (perpendicular/parallel to the maximum tumor length), sagittal T2WI, synthetic MRI, and dynamic contrast-enhanced sequence were obtained. Synthetic MRI was performed before the injection of contrast agent in the oblique axial plane, using a QRAPMASTER (quantification of relaxation times and proton density by multiecho acquisition of a saturation-recovery using turbo spin-echo readout) sequence with two echo times (19.5/97.3 ms) and four saturation delay times (210/610/1810/3810 ms). The detailed acquisition parameters are listed in Table 1. Raw data of synthetic MRI were loaded into SyMRI 8.0 (SyntheticMR, Linköping, Sweden) for postprocessing, and quantitative T1, T2, PD maps and synthetic contrast-weighted images were automatically generated within 10 s (Fig. 2).

**Table 1:** MRI parameters

	T1WI	T2WI	Synthetic MRI	DWI	DCE
Sequence	FSE	FSE	QRAPMASTER	SS-EPI	DISCO
Imaging plane	Axial	Oblique axial, oblique coronal, sagittal	Oblique axial	Axial	Axial
Repetition time (ms)	588	5115, 5912, 5062	4000	4574	4.8
Echo time (ms)	Min full	85.0, 98.8, 90.0	19.5/97.3	Minimum	2.0
Slice thickness/gap (mm)	5/1	3/0.3	3/0.3	5/1	2/0
Field of view (cm)	40 × 40	18 × 18, 24 × 24, 24 × 24	24 × 24	40 × 40	36 × 36
Matrix	320 × 256	320 × 224, 320 × 256, 320 × 224	320 × 256	128 × 128	288 × 192
Echo train length	3	24, 32, 24	16	–	–
Bandwidth (kHz)	50.00	50.00, 63.50, 50.00	27.78	250	142.86
Acquisition time (min: s)	0:57	02:39, 02:40, 2:37	04:32	01:36	Per phase 0:09

MRI, magnetic resonance imaging; T1WI, T1-weighted imaging; T2WI, T2-weighted imaging; DWI, diffusion-weighted imaging; DCE, dynamic contrast enhanced; FSE, fast spin-echo; QRAPMASTER, quantification of relaxation times and proton density by multiecho acquisition of a saturation recovery using turbo spin-echo readout; SS-EPI, single-shot echo-planar imaging; DISCO, differential sub-sampling with cartesian ordering

### Subjective image quality analysis

The subjective image quality of conventional and synthetic T2WI was evaluated by two radiologists with 5 and 8 years of experience in rectal imaging, who were blinded to pathological information and acquisition methods. The observers scored the image quality based on following 4 factors on a 5-point-Likert scale: (1) Sharpness of the lesion edge (1 = not sharp; 2 = a little sharp; 3 = moderately sharp; 4 = well sharp; 5 = very sharp); (2) Lesion conspicuity (1 = difficult to find; 2 = minimally perceivable; 3 = recognizable; 4 = easy to detect, good contrast of lesion; 5 = excellent contrast of lesion); (3) Motion artifacts (1 = severe, difficult to diagnose; 2 = a little severe, accessible to diagnose; 3 = moderate; 4 = mild; 5 = absence of artifacts); (4) Overall image quality (the three factors above added together, 1 = unacceptable; 2 = poor; 3 = moderate; 4 = good; 5 = excellent). The patient order was randomized, as was the review order of the conventional or synthetic T2WI.

### Objective image quality analysis

**Fig. 3:**
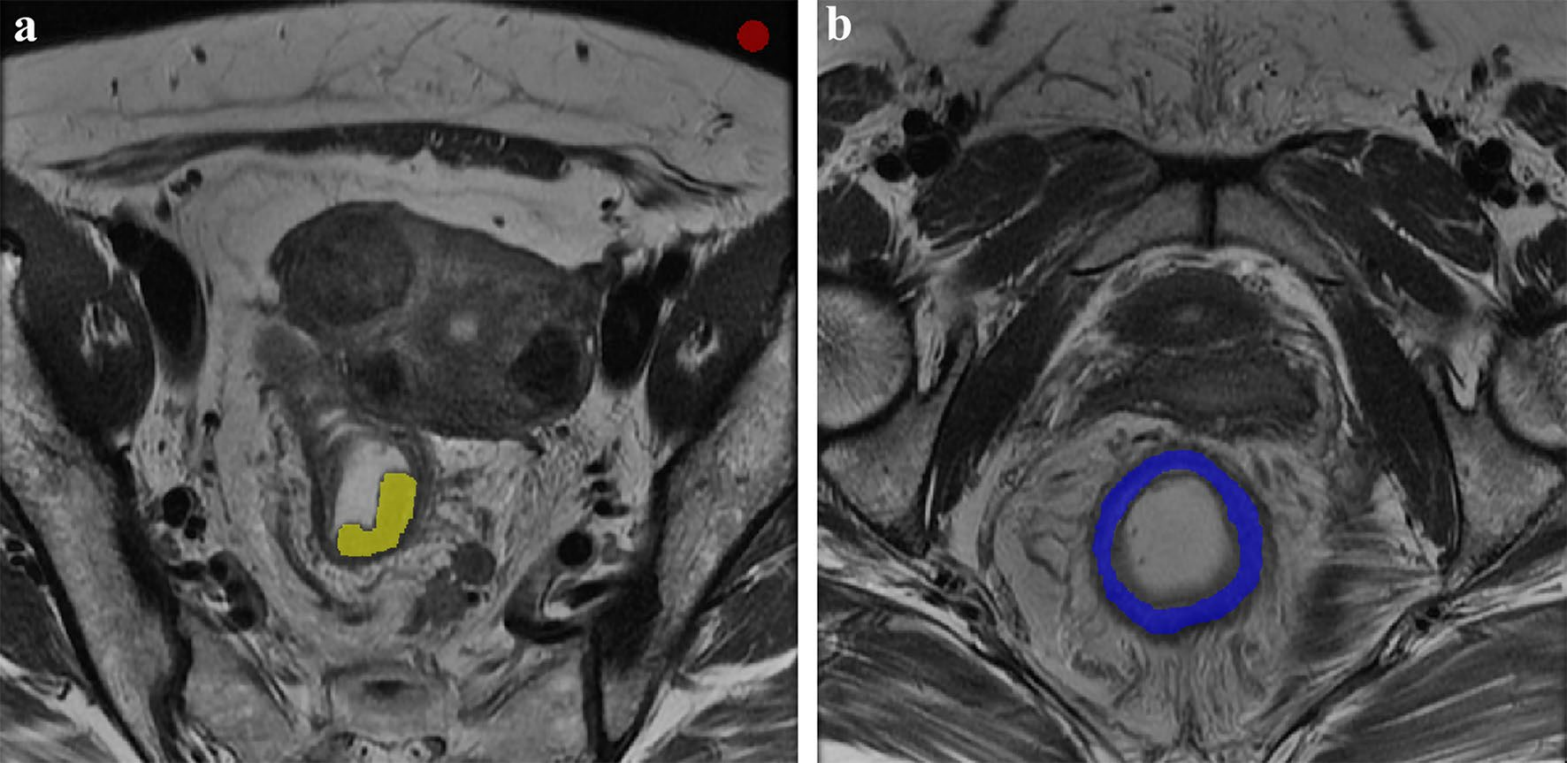
Signal-to-noise ratio (SNR) and contrast-to-noise (CNR) were calculated based on three regions of interest (ROIs). The yellow ROI represents the tumor, which was delineated slice by slice along the tumor margin. The red ROI on background was placed within the field of view but outside the body surface. The blue ROI represents normal tissue, which was drawn in normal rectum tissue at a single slice. The mean and standard deviation of the signal intensity were obtained from each ROI to calculate SNR and CNR

Regions of interest (ROI) were manually drawn on conventional oblique axial T2WI and synthetic T2WI using ITK-SNAP software (version 2.2.0, www.itksnap.org) by the same two radiologists. The ROI for the tumor was delineated on each slice along the margin of the tumor, resulting in a 3D whole tumor ROI. The normal tissue ROI was drawn in homogeneous normal rectum tissue distant from the tumor area, including the entire rectal wall at a single slice. The background ROI was a circular area with a diameter of 1 cm, placed within the field of view but outside the body surface. The mean and standard deviation of the signal intensity were obtained from each ROI. Signal-to-noise ratio (SNR) and contrast-to-noise (CNR) were calculated based on following formulas [21]:

$${\text{SNR}} = S_{{{\text{tumor}}}} /{\text{SD}}_{{{\text{background}}}} ,{\text{CNR}} = |S_{{{\text{tumor}}}} - \, S_{{{\text{tissue}}}} |{{} \mathord{\left/ {\vphantom {{} {}}} \right. \kern-\nulldelimiterspace} {}}/{\text{SD}}_{{{\text{background}}}}$$

where *S*_tumor_ is the mean signal intensity within the tumor, SD_background_ represents the standard deviation of the background noise, and *S*_tissue_ denotes the mean signal intensity of the normal tissue (Fig. 3).

### Preoperative evaluation of RC

The same two radiologists independently assessed the T stage and EMVI status on conventional and synthetic T2WI. The T stage was evaluated based on the European Society for Medical Oncology guidelines [3]. In addition, EMVI status was assessed based on a 5-point scoring system proposed by Smith et al. [22]: (0 = definitely absent; 1 = probably absent; 2 = indeterminate; 3 = probably present; 4 = definitely present). Scores of 3 or 4 were regarded as positive EMVI. Criteria for malignant node was according to the European Society of Gastrointestinal and Abdominal Radiology recommendations [23]: (1) short axis diameter ≥ 9 mm; (2) short axis diameter 5 − 8 mm and ≥ 2 morphologically suspicious characteristics; (3) short axis diameter < 5 mm and 3 morphologically suspicious characteristics; (4) all mucinous lymph nodes (any size). Morphologically suspicious criteria included round shape, irregular border, and heterogeneous signal. If there was any obvious discordance of nodal staging between two radiologists, a senior radiologist with 21 years of experience in rectal imaging made the final decision.

### Quantitative assessment

The whole tumor ROIs drawn on synthetic T2WI by two radiologists above were transferred to the quantitative maps using ITK-SNAP software. The mean T1, T2, and PD values were automatically obtained. Quantitative values acquired by the senior radiologist were used to assess the nodal staging of MRI-evaluated node-negative RC.

### Statistical analysis

The inter-observer variability for image quality score and the tumor evaluation was assessed using kappa statistics (0.21–0.40, fair; 0.41–0.60, moderate; 0.61–0.80, good; 0.81–1.00, excellent). The intraclass correlation coefficient (ICC) was used to investigate the inter-observer agreement of the SNR, CNR and quantitative values (0.21–0.40, fair; 0.41–0.60, moderate; 0.61–0.80, good; 0.81–1.00, excellent) [5]. Continuous variables were compared using the independent samples *t* test or the Mann–Whitney *U* test according to the normality of data distribution. The Wilcoxon signed-rank test was adopted to compare the image quality scores, SNR and CNR between conventional and synthetic T2WI. With pathological results as the reference standard, the differences between conventional and synthetic T2WI in evaluating the T stage and EMVI were determined using the Mc-Nemar test. Receiver operating characteristic (ROC) curves were used to assess diagnostic performance of quantitative values in nodal staging. A two-sided *p* value < 0.05 was considered statistically significant. All statistical analyses were performed using SPSS 20.0 (IBM, Armonk, NY) and MedCalc 11.4 (MedCalc, Mariakerke, Belgium).

## Results

### Clinical characteristics

All 94 patients received radical surgical resection with pathological negative circumferential resection margin. The clinical characteristics of the patients are summarized in Table 2.

**Table 2:** Clinical characteristics

Characteristics	Number of patients
Mean age, years (range)	57 (36–76)
*Gender*	
Male	66
Female	28
*Surgical resection type*	
Low anterior or anterior resection	56
Abdominoperineal resection	33
Hartman’s resection	5
*Differentiation*	
Well/Moderate	59
Poor	35
*pT stage*	
T1	6
T2	33
T3	44
T4	11
*pN stage*	
N0	59
N1	20
N2	15
*pEMVI*	
Absent	71
Present	23
*Tumor location*	
Upper	14
Middle	44
Lower	36

pEMVI, pathological extramural venous invasion

### Image quality analysis

**Fig. 4:**
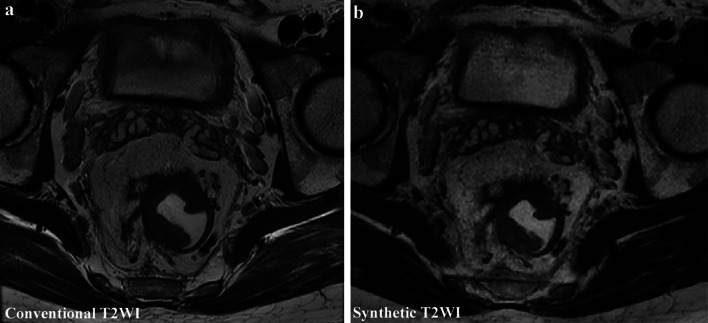
Conventional and synthetic T2-weighted imaging (T2WI) from a 46-year-old male patient with rectal cancer. Synthetic T2WI provided similar image quality with the conventional T2WI. The patient was evaluated as T3 stage with extramural venous invasion using both conventional T2WI and synthetic T2WI

The image quality scores of conventional and synthetic T2WI are shown in Table 3. There were no statistically significant differences between conventional and synthetic T2WI in SNR, CNR, overall image quality, lesion conspicuity, and absence of motion artifacts (*p* = 0.058–0.978 for all comparison pairs, Table 3). Regarding the sharpness of the lesion edge, one observer’s evaluation showed that there was no significant difference between the conventional and synthetic T2WI (*p* = 0.127), while the other observer’s evaluation had a significant difference (*p* = 0.018). Representative images from one patient are shown in Fig. 4.

**Table 3:** Comparison of image quality between conventional and synthetic T2WI

	Conventional T_2_WI	Synthetic T_2_WI	*P* value
*SNR*			
Observer 1	29.70 ± 12.17	26.85 (18.33, 41.24)	0.978
Observer 2	29.14 ± 11.94	25.85 (19.11, 41.06)	0.717
*CNR*			
Observer 1	5.41 (2.00, 9.67)	5.66 (2.89, 11.71)	0.145
Observer 2	4.99 (2.39, 9.37)	5.27 (3.10, 11.84)	0.058
*Overall image quality*			
Observer 1	4.63 ± 0.53	4.60 ± 0.54	0.592
Observer 2	4.64 ± 0.51	4.57 ± 0.54	0.239
*Lesion conspicuity*			
Observer 1	4.69 ± 0.53	4.71 ± 0.52	0.617
Observer 2	4.65 ± 0.56	4.66 ± 0.54	0.808
*Sharpness of the lesion edge*			
Observer 1	4.59 ± 0.50	4.69 ± 0.46	0.018*
Observer 2	4.64 ± 0.51	4.71 ± 0.46	0.127
*Absence of motion artifacts*			
Observer 1	4.68 ± 0.53	4.56 ± 0.58	0.069
Observer 2	4.64 ± 0.55	4.53 ± 0.58	0.112

SNR and CNR following the normal distribution are expressed as mean ± standard deviation; otherwise, expressed as median (first quartile, third quartile). The subject image scores are expressed as means ± standard deviations

SNR, signal-to-noise ratio; CNR, contrast-to-noise ratio

*p < 0.05

### Evaluation of T stage and EMVI

Comparisons of the diagnostic accuracy of T stage and EMVI using conventional and synthetic T2WI are presented in Table 4. With pathological T stage as the reference standard, there were no significant differences in the diagnostic accuracy of T stage between conventional and synthetic T2WI from both observers (*p* = 0.375 and 0.625). Similarly, the differences in the accuracy for evaluating EMVI between conventional and synthetic T2WI were not statistically significant from both observers (*p* = 0.625 and 0.219).

**Table 4:** Comparison of diagnostic accuracy of T stage and EMVI using conventional and synthetic T2WI

	Conventional T2WI	Synthetic T2WI	*P* value
*mrT stage*			
Observer 1	90.4% (85/94)	85.1% (80/94)	0.375
Observer 2	91.5% (86/94)	87.2% (82/94)	0.625
*mrEMVI*			
Observer 1	81.9% (77/94)	79.8% (75/94)	0.625
Observer 2	85.1% (80/94)	78.7% (74/94)	0.219

Numbers used to calculate percentages are in parentheses. mrT stage, T stage on T2-weighted imaging; mrEMVI, extramural venous invasion on T2-weighted imaging

### Quantitative assessment

**Fig. 5:**
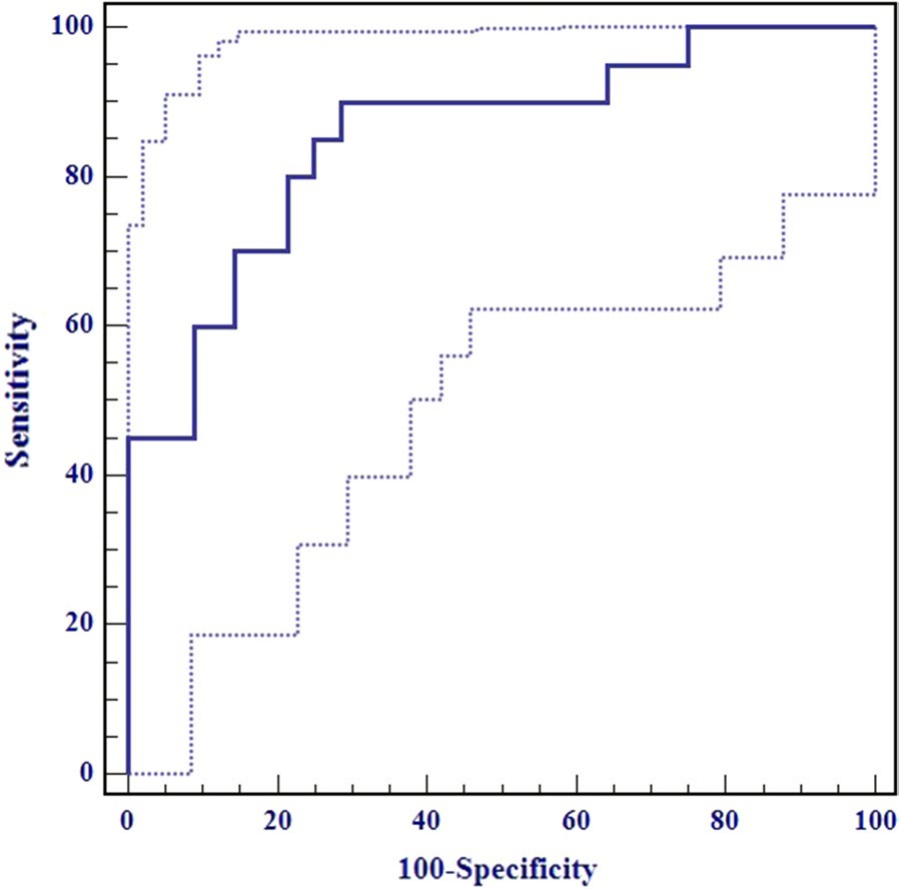
Receiver operating characteristics (ROC) curve of the T2 value for predicting nodal staging of preoperatively node-negative rectal cancer with the area under the ROC, sensitivity, specificity, and accuracy of 0.854, 90.0%, 71.4%, and 80.3%, respectively. The solid line represents the area under the ROC curve. The dotted line represents the 95% confidence interval

Among the 76 patients without suspicious metastatic lymph node on preoperative MRI, 56 were pathologically confirmed pN0 stage, and 20 were pN1-2. The differences in the T1, T2 and PD values between the pN0 and pN1-2 groups are listed in Table 5. The T2 values of the pN1-2 group were significantly lower than those of the pN0 group (*p* < 0.001). The T2 value demonstrated good diagnostic performance for predicting nodal staging of preoperatively node-negative RC with the area under the ROC (AUC), sensitivity, specificity, and accuracy of 0.854 (95% confidence interval, CI, 0.755 − 0.925), 90.0%, 71.4%, and 80.3%, respectively. The ROC curve is shown in Fig. 5.

**Table 5:** Differences of quantitative parameters between the pN0 and pN1-2 groups

Parameters	pN0 (n = 56)	pN1-2 (n = 20)	*P* value
T1 (ms)	1492.47 (1437.33, 1619.47)	1492.62 ± 173.93	0.273
T2 (ms)	95.15 ± 4.60	87.44 ± 5.66	< 0.001*
PD (pu)	61.45 (54.65, 65.91)	60.44 ± 9.017	0.860

Data following the normal distribution are expressed as mean ± standard deviation. Otherwise, data are expressed as median (first quartile, third quartile)

PD, proton density

*p < 0.05

### Interobserver reliability

The results of interobserver agreements are summarized in Additional file 1: Table S1. The SNR and CNR of conventional and synthetic T2WI all demonstrated excellent interobserver agreements (ICC > 0.9). The interobserver agreements of subjective image quality scores for conventional and synthetic T2WI were good or excellent (kappa values = 0.800–0.957). The interobserver agreements of mrT stage and mrEMVI were all excellent for conventional and synthetic T2WI (kappa values = 0.892 and 0.810 for mrT stage; 0.854 and 0.865 for mrEMVI). The T1, T2, and PD value demonstrated excellent interobserver agreement (ICC = 0.916, 0.953, and 0.973, respectively).

## Discussion

We preliminarily investigated the feasibility of using synthetic MRI for the preoperative evaluation of RC. Synthetic T2WI provides similar image quality with the conventional T2WI, and achieves comparable diagnostic accuracy in assessing T stage and EMVI in RC patients. Additionally, T2 value demonstrated good diagnostic performance of nodal staging of MRI-evaluated node-negative patients.

The main advantage of synthetic MRI is the simultaneous generation of multiple images in a single scan, including quantitative relaxation mappings and synthetic morphological contrast-weighted images. The efficiency of synthetic MRI in the clinical application may depend on whether the total acquisition time is reduced and image quality is favorable. The acquisition time of synthetic MRI in our study was similar to that of T2 mapping in previous studies [9, 24], while synthetic MRI could simultaneously provide information of T1 mapping, PD mapping, T2WI, etc. Therefore, the scanning time of synthetic MRI is less than that with separately acquired contrast-weighted images and quantitative relaxation mappings.

Another primary issue is to ensure the image quality of synthetic MRI for further extensive clinical application. Our results demonstrated that the image quality scores of synthetic T2WI were comparable to that of conventional T2WI in terms of the SNR, CNR, overall image quality, lesion conspicuity, and the absence of motion artifacts. For the sharpness of the lesion edge, synthetic T2WI was rated superior to that of the conventional T2WI by one observer, which may be related to the observers’ interpreting experience. More experienced observers may be used to conventional images. Furthermore, there was overall good to excellent interobserver agreement among the image quality scores for both conventional and synthetic T2WI. Therefore, we preliminarily speculated that synthetic T2WI could achieve similar image quality to that of conventional T2WI.

To further confirm the clinical feasibility of synthetic MRI, we evaluated T stage and EMVI using synthetic T2WI and conventional T2WI. The diagnostic accuracy of EMVI by two observers was consistent with previous studies [25–27], as was the T stage [28–30]. Although the diagnostic accuracy with conventional T2WI was slightly higher than that with synthetic T2WI, the difference was not statistically significant. It may be due to the fact that the signal intensity of synthetic T2WI was generally lower than that of conventional T2WI [14, 31], and observers are more accustomed to the contrast and signal intensity of conventional T2WI. Therefore, we proposed that, with training and adaptation, synthetic T2WI might be as suitable for T stage and EMVI assessment of RC as conventional T2WI.

Our previous study confirmed the value of quantitative relaxation mapping in evaluating prognostic factors of RC, in which the potential of the T2 value for predicting RC nodal staging has been demonstrated [17]. Some RC patients with micro-nodal involvement may be underestimated as node-negative at initial MRI, so this study focused on these patients. A previous study established a nomogram based on clinical factors for predicting nodal staging in clinically node-negative RC patients with an AUC of 0.743 [32]. In our study, the T2 value demonstrated good diagnostic performance for predicting nodal staging of preoperatively node-negative RC with an AUC of 0.854 (95% CI 0.755–0.925), which was superior to the previous study. We speculated that the T2 value could provide additional information for improving nodal staging of MRI-evaluated node-negative patients, which was beneficial to correctly enroll RC patients for neoadjuvant treatment.

There were some limitations in this study. Firstly, this was a single-center study with a relatively small sample size. Further prospective multi-center studies with larger sample sizes are warranted to validate our preliminary foundings. Secondly, we only evaluated the image quality of T2WI generated by synthetic MRI and did not evaluate other images. At present, high resolution T2WI is mainly used in preoperative evaluation of RC, while the application value of other non-contrasted enhanced images such as T1WI is limited. Therefore, evaluating the image quality of other contrast-weighted images is of little clinical value. Finally, we did not evaluate all tumor characteristics including sphincter invasion, and quantitative analysis of lymph node was not performed in this preliminary study. These aspects will be included in further prospective studies.

## Conclusions

Synthetic MRI may facilitate preoperative staging and EMVI evaluation of RC by providing synthetic T2WI and quantitative maps in one acquisition.

## Supplementary Information

**Additional file 1.** Interobserver agreement of imaging quality score, mrT stage, and mrEMVI.

## Data Availability

The datasets used and/or analyzed during the current study are available from the corresponding author on reasonable request.

## Abbreviations

CI: Confidence interval
CNR: Contrast-to-noise
EMVI: Extramural venous invasion
ICC: Intraclass correlation coefficient
PD: Proton density
RC: Rectal cancer
ROC: Receiver operating characteristic
ROI: Regions of interest
SNR: Signal-to-noise ratio
T2WI: T2-weighted imaging
