# Supplementary material for: A preliminary study of synthetic magnetic resonance imaging in rectal cancer: imaging quality and preoperative assessment
Source: Insights Imaging. 2021 Aug 21;12:120. doi: 10.1186/s13244-021-01063-w (PMC8380206; doi:10.1186/s13244-021-01063-w)
Supplement: Supplementary file 1 — Additional file 1. Interobserver agreement of imaging quality score, mrT stage, and mrEMVI. [file 13244_2021_1063_MOESM1_ESM.doc]

**ELECTRONIC SUPPLEMENTARY MATERIAL**

Table S1. Interobserver agreement of image quality score, mrT stage, and mrEMVI

| ICC or kappa value | Conventional T2WI | Synthetic T2WI |
| --- | --- | --- |
| SNR (ICC) | 0.969 (0.954 - 0.980) | 0.978 (0.967 - 0.985) |
| CNR (ICC) | 0.956 (0.934 - 0.971) | 0.939 (0.909 - 0.959) |
| Overall image quality (kappa) | 0.932 | 0.957 |
| Lesion conspicuity (kappa) | 0.853 | 0.874 |
| Sharpness of the lesion edge (kappa) | 0.800 | 0.949 |
| Absence of motion artifacts (kappa) | 0.857 | 0.938 |
| mrT stage (kappa) | 0.892 | 0.810 |
| mrEMVI (kappa) | 0.854 | 0.865 |

Note: ICC, intraclass correlation coefficient; SNR, signal-to-noise ratio; CNR, contrast-to-noise ratio; mrEMVI, extramural venous invasion on MRI. SNR and CNR were evaluated by ICC (95% confidence interval) and others by the kappa value.
